# Adenoviral E1A Exploits Flexibility and Disorder to Target Cellular Proteins

**DOI:** 10.3390/biom10111541

**Published:** 2020-11-11

**Authors:** Maria Grazia Murrali, Isabella C. Felli, Roberta Pierattelli

**Affiliations:** Department of Chemistry “Ugo Schiff” and Magnetic Resonance Center (CERM), University of Florence, Via Luigi Sacconi 6, 50019 Sesto Fiorentino (Florence), Italy; mgmurrali@gmail.com

**Keywords:** E1A, CBP, NMR, IDP, fuzzy complex

## Abstract

Direct interaction between intrinsically disordered proteins (IDPs) is often difficult to characterize hampering the elucidation of their binding mechanism. Particularly challenging is the study of fuzzy complexes, in which the intrinsically disordered proteins or regions retain conformational freedom within the assembly. To date, nuclear magnetic resonance spectroscopy has proven to be one of the most powerful techniques to characterize at the atomic level intrinsically disordered proteins and their interactions, including those cases where the formed complexes are highly dynamic. Here, we present the characterization of the interaction between a viral protein, the Early region 1A protein from Adenovirus (E1A), and a disordered region of the human CREB-binding protein, namely the fourth intrinsically disordered linker CBP-ID4. E1A was widely studied as a prototypical viral oncogene. Its interaction with two folded domains of CBP was mapped, providing hints for understanding some functional aspects of the interaction with this transcriptional coactivator. However, the role of the flexible linker connecting these two globular domains of CBP in this interaction was never explored before.

## 1. Introduction

**Figure 1:**
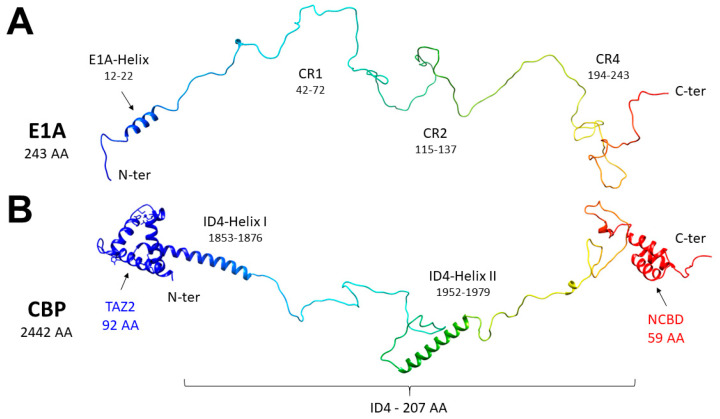
Structural features of (**A**) human adenovirus E1A12S and (**B**) human CBP-ID4. For the latter, also the flanking domains CBP-TAZ2 and CBP-NCBD are displayed for illustrative purposes. The two models were built as described in the experimental section.

The adenovirus (AdV) is responsible for respiratory and gastric infections in humans and oncogenic transformation in rodents. The early region 1A (E1A) gene is the first expressed following adenoviral infection and it plays a central role in viral function, activating the genes required for viral replication and interfering with the host cell cycle regulation [1,2]. The E1A gene encodes two major proteins of 243 and 289 amino acids (E1A12S and E1A13S, respectively) early after infection. The sequence comparison of the E1A13S of several adenovirus serotypes displays the presence of four conserved regions, termed CR1 to CR4, which are separated by less conserved regions [3,4]. E1A13S and E1A12S are identical except for the CR3, a module of 46 amino acids, which belongs only to the larger of the two. The regions considered relevant for both variants are the N-terminus (aa 1–41), the CR1 (aa 42–72), the CR2 (aa 115–137), and the CR4 at the C-terminus (Figure 1A). The two E1A splicing variants were characterized by NMR spectroscopy, showing that they are both highly disordered [5]. Only the CR3 region presents a minimal fold composed by two α-helices of less than 10 amino acids each, embedded in a highly flexible polypeptide [5]. The four conserved regions are involved in multiple interactions with other proteins to regulate the AdV transcription and cell cycle control [6]. CR1 and CR2, which are in common between the two E1A proteins, are responsible for driving inhibited primary cells into S Phase, accounting for the oncogenic transformation of cells in rodents [7]. The CR3 influences the viral transcription by interacting with the super elongation complex (SEC) of RNA Polymerase II via the multisubunit mediator complex, to promote the transcriptional elongation on a free viral genome [8].

AdV, like any other virus, is an obligate intracellular parasite completely dependent on host cell functions. By targeting multiple cellular hubs, AdV can virtually reprogram all mechanisms of cell biology, rendering the infected cell an amenable environment for virus replication [9]. The high flexibility of E1A, able to adopt multiple conformations and to bind to diverse partners maximizing interaction capabilities [10], is crucial for its function. E1A participates in important protein–protein interactions with over 50 cellular factors, deregulating the cell cycle by interfering with key cellular proteins [2]. One of the most important, and most studied, interactions of E1A is the one with the CREB-Binding Protein (CBP) and its paralog p300 [11]. CBP/p300 is a transcriptional coactivator [12], able to interact with a large number of proteins through multiple domains and with acetyl-transferase activity, playing critical roles in basic cellular processes [13]. This 2442 residue-long protein presents seven folded domains that have been structurally characterized in recent years, either through X-ray or NMR [14,15,16,17,18]. These are separated by five disordered linkers (denoted ID#, where # is the number of the linker) [19]. Interestingly, the regions outside the globular domains are predicted to be intrinsically disordered, constituting nearly 60% of the protein composition. Thanks to recent progress in NMR spectroscopy, three of the five disordered linkers have been structurally characterized at atomic resolution [19,20,21], but little is known about their functional roles.

The interaction between E1A and CBP has been reported in several studies [22,23] revealing that two-folded domains of CBP [2,13,24], the transcriptional adaptor zinc finger-2 domain (TAZ2) and the nuclear coactivator binding domains (NCBD), are the main responsibles for binding. These globular domains of CBP involved in the interaction with E1A are connected by a flexible linker (CBP-ID4, residues 1851–2057 of human CBP, 207 aa), considered until recently just as a unit connecting the otherwise functional globular domains, despite its length (Figure 1B).

The TAZ2 domain binds specifically to the acidic transactivation domains of several transcription factors, including the p53 tumour suppressor ETF2 and members of the STAT and C/EBP families [25,26,27]. The interaction of E1A with this domain competes with these interactions and it can inhibit the histone acetyltransferase activity. This can also prevent interactions of CBP with other transcription factors. The association of E1A with CBP/p300 results in the global hypoacetylation of K18 of H3 and may be linked to the ability of E1A to induce oncogenic transformations [1]. Moreover, E1A can compete with cellular factors for binding to NCBD, being able to repress differentiation and the immune response to viral infection [18].

The interaction of fragments of E1A with the CBP-TAZ2 and CBP-NCBD single domains was investigated by NMR spectroscopy [28,29]. Two fragments of E1A encompassing the N-terminus (E1A_1–36_) and the CR1 (E1A_53–91_), respectively, were selected to study the interaction with TAZ2. The binding of E1A_1–36_ is in intermediate exchange on the NMR timescale, while binding of E1A_53–91_ has higher affinity and is in slow exchange on the NMR timescale [28]. Lately, binding affinities were measured by monitoring changes in intramolecular FRET that accompany the folding of E1A upon binding to TAZ2, obtaining a K_d_ = 11.7 nM for a construct encompassing residues 1–101 [30]. Similarly, NMR was used to investigate the interaction of E1A with NCBD. In this case, it was possible to estimate the binding affinity for the fragment E1A_53–91_ by NMR spectroscopy titration, obtaining a K_d_ = 1.0 μM for the N-terminus of E1A and K_d_ = 75 μM for E1A_53–91_ [29]. The interaction of the fragments of E1A with NCBD is weaker than the one with TAZ2, forming complexes that are in fast exchange with the isolated form on the NMR timescale.

Can the linker between CBP-TAZ2 and CBP-NCBD be an additional target for E1A? To advance our understanding of the mechanism through which E1A interferes with CBP function and the possible role of targeting its disordered domains, we investigated the interaction between E1A12S and the CBP-ID4 linker.

## 2. Materials and Methods

The ^15^N-labeled and unlabeled E1A12S [5], the ^15^N labeled, ^13^C^15^N labeled and unlabeled CBP-ID4 [19] were prepared as previously described. The NMR samples were prepared exchanging the final buffer in a degassed water buffer containing 20 mM TRIS, 50 mM KCl, 1 mM TCEP at pH 7.4, with 10% D_2_O added for lock signal. 

For all the titrations, the solutions of labeled proteins were split equally into two samples. One of these was mixed with the unlabeled titrant to obtain a 1:2 complex and the other was mixed with the same volume of NMR buffer. These two solutions were used to prepare the various samples with different molar ratios of the two proteins. Five points per titration were recorded. 

The isolated form of 100 µM ^15^N E1A12S and the 1:1 complex of ^15^N E1A12S and CBP-ID4 with a final buffer containing 20 mM TRIS, 1 mM TCEP at pH 7.4 were prepared with different salt concentrations (50 mM KCl, 150 mM KCl, and 300 mM KCl). 

The ^1^H-^15^N FHSQC [31] and ^1^H-^15^N BEST-TROSY [32] spectra were acquired at 283 K on a 22.3 T Bruker AVANCE III NMR spectrometer (Bruker BioSpin AG, Fällanden, Switzerland), operating at 950 MHz ^1^H, 238.8 MHz ^13^C, and 96.32 MHz ^15^N equipped with an inverse triple resonance cryogenically cooled probehead (TCI) in 3 mm NMR tubes.

The ^13^C detected experiments were acquired at 16.4 T Bruker AVANCE NEO NMR spectrometer (Bruker BioSpin AG, Fällanden, Switzerland), operating at 700.06 MHz ^1^H, 176.05 MHz ^13^C, and 70.97 MHz ^15^N equipped with a direct triple-resonance cryogenically cooled probehead optimized for ^13^C detection (TXO). Titration points were recorded in 5 mm Shigemi NMR tubes, for 2D CON [33], 2D H^α^-CON^pro^ [34], 2D H^N^-CON [35].

All the heteronuclear relaxation experiments (^15^N R_1_, ^15^N R_2,_ and ^1^H–^15^N NOE) [36,37,38] were acquired using 150 µM E1A12S and E1A12S + CBP-ID4 1:1 complex or 200 µM CBP-ID4 and CBP-ID4 + E1A12S 1:1 complex. The spectra were recorded at 283 K on a 16.4 T Bruker AVANCE NEO 700 spectrometer NMR (Bruker BioSpin AG, Fällanden, Switzerland) operating at 700.13 MHz ^1^H, 176.05 MHz ^13^C, and 70.94 MHz ^15^N equipped with an inverse triple resonance cryogenically cooled probehead (TCI).

The ^15^N R_1_ and R_2_ experiments were acquired with 8 scans per increment (2048 × 256 points) and a relaxation delay of 3.0 s. To determine the ^15^N R_1,_ the following delays were used: 20, 60, 120, 180, 240, 400, 500, 600, 800 ms, and 1.0 as well as 1.2 s. To determine the ^15^N R_2,_ the following delays were used: 32, 64, 96, 128, 160, 190, 260, 320, 380, 440, and 500 ms. The ^1^H–^15^N NOEs experiments were acquired with 48 scans (2048 × 256 points) and a relaxation delay of 6.0 s.

The ^15^N relaxation rates (R_1_ and R_2_) were determined by fitting the cross-peak intensities measured as a function of the variable delay, to single-exponential decay curves using the Bruker Dynamic Center 2.4, available as a stand-alone ancillary software of TopSpin by Bruker. ^1^H-^15^N NOE values were obtained as the ratio between peak intensities in spectra recorded with and without ^1^H saturation. All the spectra were acquired, processed, and analyzed by using Bruker TopSpin 3.5pl7 or Bruker TopSpin 4.0.1 software. The spectra were analyzed and annotated using the program CARA [39].

Circular dichroism spectra to assess the stability of the protein secondary structure were acquired in the far-UV with a Jasco J-810 spectropolarimeter (Jasco Europe S.R.L.,Italy) using a 0.1 cm path length quartz cuvette. The mean of 5 scans between 190 and 250 nm wavelength was calculated by subtraction of the corresponding buffer spectrum. The sample concentration was 30 µM.

Structural models were created with Flexible-Meccano [40] using standard parameters and secondary structure propensities previously determined [5,19]. For the cartoons in Figures 1 and 9, a conformer of E1A and a conformer of CBP-ID4 were arbitrarily selected; the latter was merged with the TAZ2 (PDB ID 2KJE) and NCBD (PDB ID 2KKJ) models. The models were displayed by using UCSF Chimera [41], a molecular graphics program developed by the Resource for Biocomputing, Visualization, and Informatics at the University of California, San Francisco.

## 3. Results

**Figure 2:**
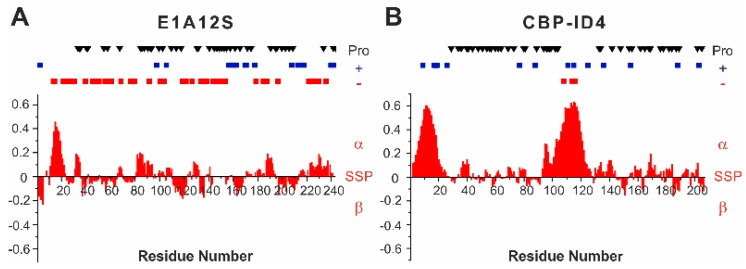
**** Secondary structure propensity of E1A12S (**A**) and CBP-ID4 (**B**). Proline residues are highlighted along the protein sequence as black triangles. Positive residues are highlighted as blue squares, negative residues are highlighted as red squares.

E1A12S was characterized at high resolution by NMR spectroscopy [5] and found to be highly disordered, with a mild propensity to sample the helical conformation in the N-terminal part of the protein, in the region encompassing residues 12–22 (Figure 2A). The amino acids sequence is rich in negatively charged residues (30 Glu and 14 Asp), which are distributed all along the sequence, with two highly negatively charged fragments in the region 130–155 (^133^DDEDEEGEE^141^ and ^145^EPEPEPEPEP^154^). Positively charged residues (14 Arg and 3 Lys) are mainly located in the second half of the polypeptide. The 43 proline residues, which constitute 18% of the aminoacids, are distributed along the sequence, except for the N-termius region harboring the partially populated helical region.

The CBP-ID4 linker has also been characterized by NMR spectroscopy [19]. It is largely disordered but presents two partially populated helical fragments termed Helix I (2–25 aa, residues 1852–1875 of CBP) and Helix II (101–128 aa, residues 1951–1978 of CBP) (Figure 2B). These segments are well conserved across the species, suggesting their possible functional role [42]. The rest of the polypeptide is interspersed by proline residues, which are 45 in total (22%). The overall charge of the protein is positive (1 Lys and 13 Arg), with very few negative residues located in the second helical region (3 Glu).

### 3.1. Mapping the Interaction between E1A12S and CBP-ID4

**Figure 3:**
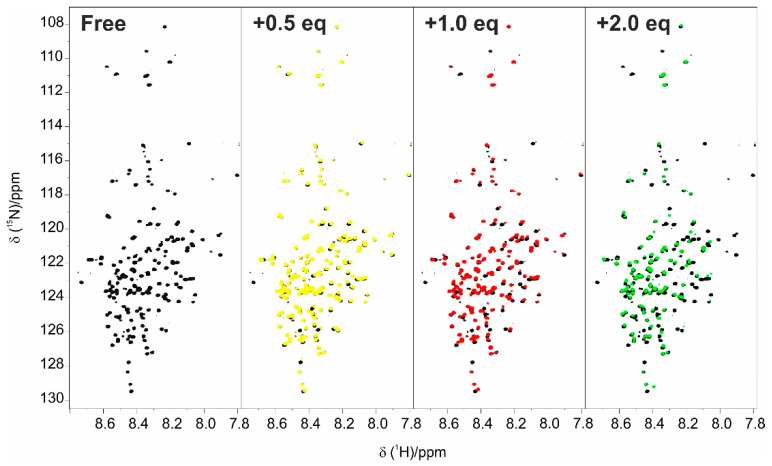
**** Titration of E1A12S with CBP-ID4 followed by ^1^H-^15^N BEST-TROSY experiments. E1A12S (black) E1A12S:CBP-ID4 at 1:0.5 molar ratio (yellow); E1A12S:CBP-ID4 at 1:1 molar ratio (red); and E1A12S:CBP-ID4 at 1:2 molar ratio (green). All the experiments were acquired at 283 K, using a 22.3 T Bruker Avance III NMR spectrometer equipped with a TCI CryoProbe^TM^.

The first step for the characterization of the interaction of the two proteins was the titration of E1A12S with CBP-ID4. The addition of increasing amounts of unlabelled CBP-ID4 to ^15^N-labelled E1A (Figure 3) caused several changes in the ^1^H-^15^N BEST-TROSY spectrum of E1A. In particular, the first half of the sequence of E1A is perturbed by the addition, showing different interaction modalities. The NMR signals belonging to residues in this region showed variation in the chemical shift and broadening due to exchange regimes between the free and bound state that ranged from fast to intermediate on the NMR time scale. 

**Figure 4:**
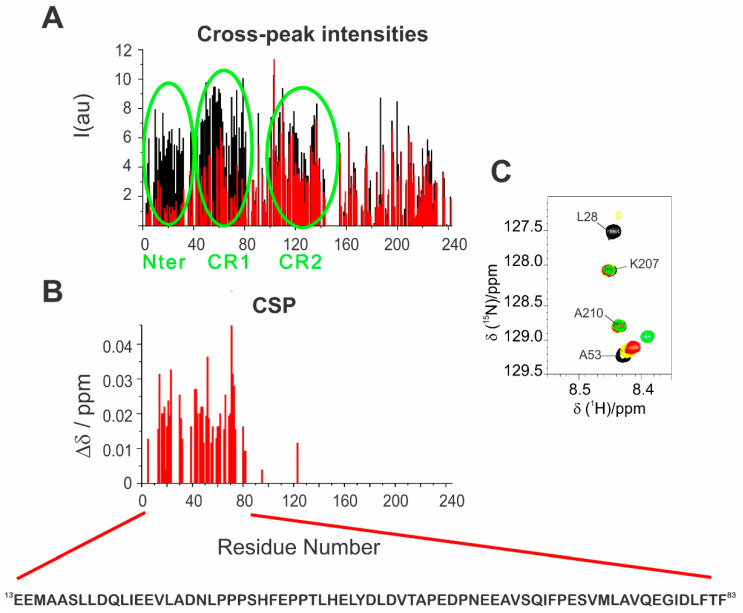
(**A**) Comparison of the cross-peak intensities in ^1^H-^15^N BEST-TROSY experiments for E1A12S in the isolated form (black) and E1A12S:CBP-ID4 1:1 complex (red); (**B**) mapping the chemical shift perturbations of E1A12S signals in the E1A12S:CBP-ID4 1:1 complex using the Garrett equation; (**C**) comparison of a selected region of ^1^H-^15^N BEST-TROSY spectrum of E1A12S (black), E1A12S:CBP-ID4 1:0.5 (yellow), E1A12S:CBP-ID4 1:1 (red), and E1A12S:CBP-ID4 1:2 molar ratio (green) with signals assignment.

The two effects described above are more pronounced for the first half of the primary sequence with a prominent effect in two regions: 2–32 (N–terminus) and 66–83 (CR1) (Figure 4A), as also confirmed by chemical shift perturbations observed for the residues in fast exchange, as shown in the Garrett plot [43,44] for the 1:1 complex (Figure 4B,C). In addition, a small fraction of peaks of residues in the CR2 region showed a decrease in intensity upon interaction.

**Figure 5:**
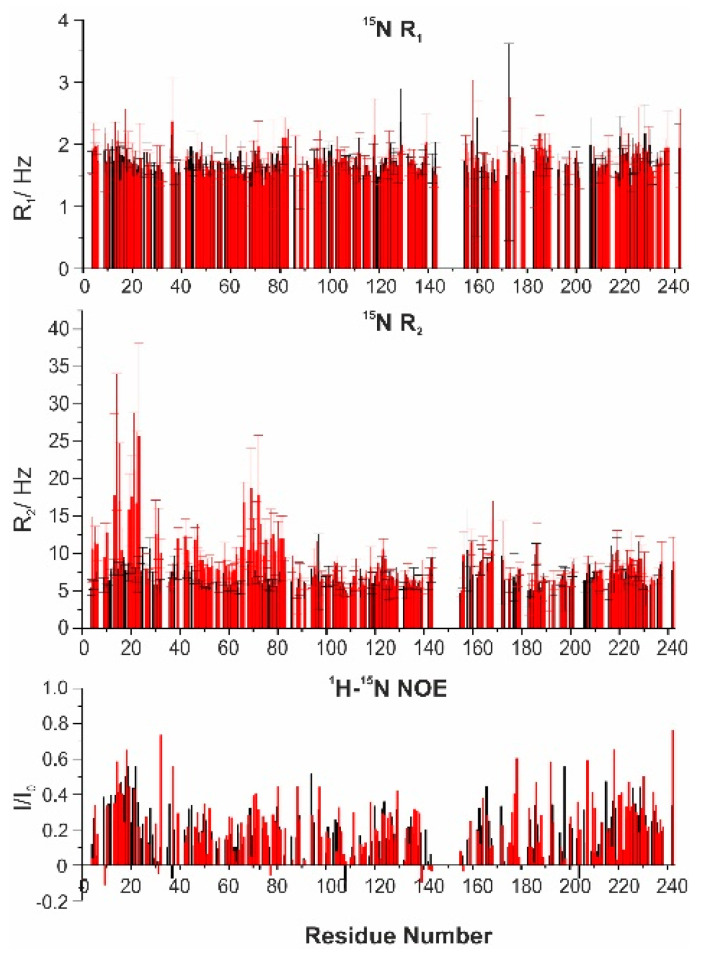
Nuclear relaxation data for the backbone amide ^15^N obtained for E1A12S (black) and the 1:1 complex E1A12S:CBP-ID4 (red). From top to bottom, ^15^N longitudinal relaxation rates (R_1_, Hz), ^15^N transverse relaxation rates (R_2_, Hz) and ^1^H-^15^N NOE values. In the plot, the missing data derive from residues that are not observable or not resolved.

On the 1:1 adduct, ^15^N R_1_ and R_2_ relaxation rates and ^1^H-^15^N NOE values were measured (Figure 5) and compared with those of the free protein. The R_2_ values determined for the residues in the first part of the protein were significantly higher for the complex than for the isolated protein, and in particular for the residues 9–39 and 65–80, while the rest of the polypeptide was unaffected by the interaction. The ^1^H-^15^N NOE and the R_1_ values were only slightly varied.

As noted above, both E1A12S and CBP-ID4 have a large number of charged residues, resulting in opposite isoelectric points (pI), with E1A12S that can be considered an acidic protein with pI 4.4 and CBP-ID4 that is a basic protein with pI 12.2. Indeed, the interaction is affected by the ionic strength of the solution: the increase in the ionic strength of the solution from 50 to 300 mM KCl (Figure S1) causes a reduction in the chemical shifts variations due to the interaction between the two proteins, but the effect for the signals in the N-terminus is still evident, in particular for residues 14 to 40. 

The region of E1A mostly perturbed upon the addition of CBP-ID4 (1–85) is rich in negatively charged (about 20% of the amino acids) and hydrophobic amino acids (with Ile, Val and Leu accounting for 26% or the amino acids) as well as in Pro (12%). However, looking in detail into the primary sequence reveals peculiar characteristics in the regions that are perturbed to different extents: the first region (2–32) is very rich in large, hydrophobic amino acids and lacks proline residues, in agreement with the presence of a partially populated α-helix in the fragment 12–22; the second region (33–64) is very rich in proline residues; the third one (66–85), highly perturbed upon interaction with CBP-ID4, also contains two phenylalanine-proline pairs (66–67 and 83–84). The latter (aromatic-proline pairs) have been shown to play an important role in promoting the formation of compact states in intrinsically disordered proteins IDPs and might also have a relevant role in mediating interactions with partners [45]. Indeed as also noticed earlier [28], a little cluster of hydrophobic residues is present in the region 67–72, well conserved among the various AdV variants, suggesting a functional role for this region in recruiting other proteins. This region is one of the most affected by interaction with CBP-ID4, and we can envisage that this interaction is instrumental for E1A to target CBP. Finally, it is interesting to note that the region with the highest density of negative charges (^133^DDEDEEGEE^141^ and ^145^EPEPEPEPEP^154^) is also slightly perturbed but to a lower extent with respect to the other ones mentioned above, indicating that although largely driven by electrostatics, the interaction is also driven by the involvement of hydrophobic amino acids (Ile, Val, Leu as well as Phe in the final part).

### 3.2. Mapping the Interaction between CBP-ID4 and E1A12S

**Figure 6:**
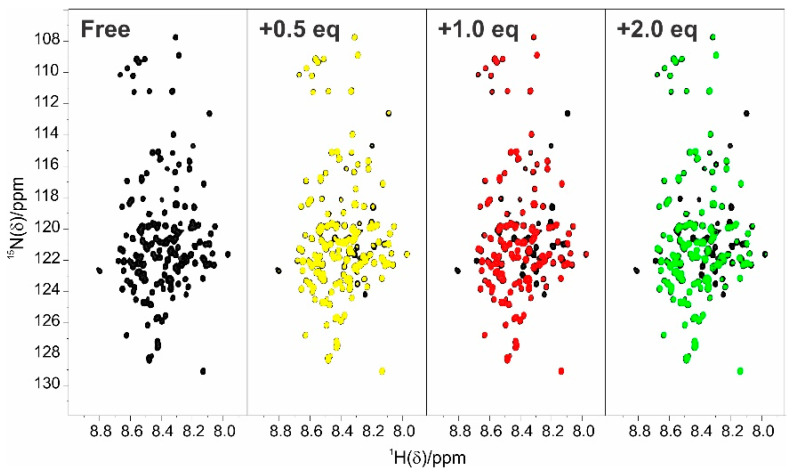
Titration of CBP-ID4 with E1A12S followed by ^1^H-^15^N FHSQC experiments. CBP-ID4 (black); CBP-ID4:E1A12S at 1:0.5 molar ratio (yellow); CBP-ID4:E1A12S at 1:1 molar ratio (red); and CBP-ID4:E1A12S at 1:2 molar ratio (green). All the experiments were acquired at 283 K, using a 22.3 T Bruker Avance III NMR spectrometer equipped with a TCI CryoProbe^TM^.

The titration of ^15^N CBP-ID4 with unlabelled E1A12S showed no chemical shift perturbations of the CBP-ID4 signals in ^1^H-^15^N correlation spectra, but several signals disappear or lose intensity progressively by increasing the concentration of E1A12S (Figure 6).

Most of the peaks absent in the spectrum of the adduct belong to the first part of the protein, suggesting a possible involvement of the first α-helical region in the interaction. The overlap in the HN-correlation spectra, however, prevented a detailed description.

To better characterize the binding site, we exploited ^13^C detection experiments [46], which provide an increased dispersion of nuclear chemical shifts and straightforwardly enable the detection of proline residues [34]. The simplest ^13^C-detected experiment is the 2D CON that allows detecting the signals that correlate the backbone carbonyl carbon of one amino acid (C’_i_) with the amide nitrogen of the neighboring amino acid involved in the peptide bond (N_i+1_) [47]. To acquire the C’_i_-N_i+1_ correlation, several experimental variants can be used, exploiting C’, H^α^, or H^N^ as a starting magnetization source, which present different features in terms of sensitivity, acquisition time, and detected signals [33,35,48]. 

**Figure 7:**
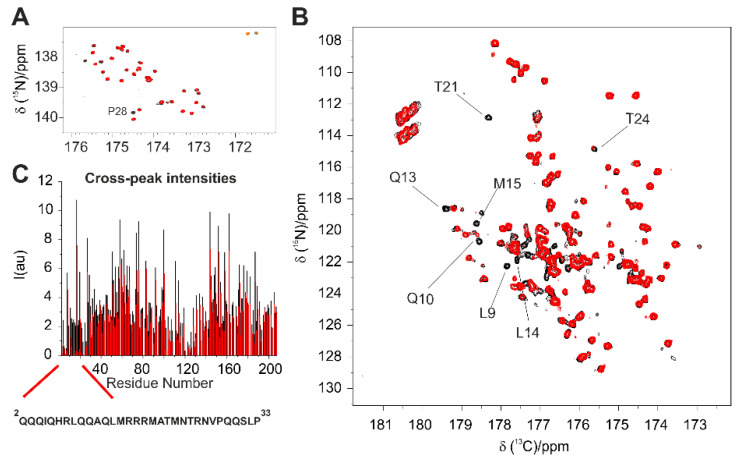
(**A**) Comparison of 2D H^α^-CON^pro^ spectra of CBP-ID4 (black) and CBP-ID4:E1A12S 1:1 (red); (**B**) comparison 2D H^N^-CON spectra of CBP-ID4 (black) and CBP-ID4:E1A12S 1:1 (red). The assignment of selected peaks is also reported. (**C**) Comparison of cross-peak intensities in 2D H^N^-CON and 2D H^α^-CON^pro^ experiments for CBP-ID4 in the isolated form (black) and CBP-ID4:E1A12S 1:1 complex (red). All the spectra were acquired at 283 K with a 16.4 T Bruker Avance NEO NMR spectrometer equipped with a ^13^C TXO CryoProbe^TM^.

In the C’-start 2D CON of the 1:1 adduct (not shown), the proline region reveals that most of them are not perturbed upon interaction, except Pro 28, which is located at the end of Helix I, as confirmed with a 2D H^α^-CON^pro^ experiment [34] focusing on this region (Figure 7A); a large number of peaks belonging to residues in the two α-helical regions instead could not be detected. However, exploiting the more sensitive H^N^ CON, it became evident that most of the peaks affected by the interaction belong to residues in the Helix I, while most peaks of residues in Helix II remain unchanged (Figure 7B). These results confirm Helix I as the main target of E1A. 

**Figure 8:**
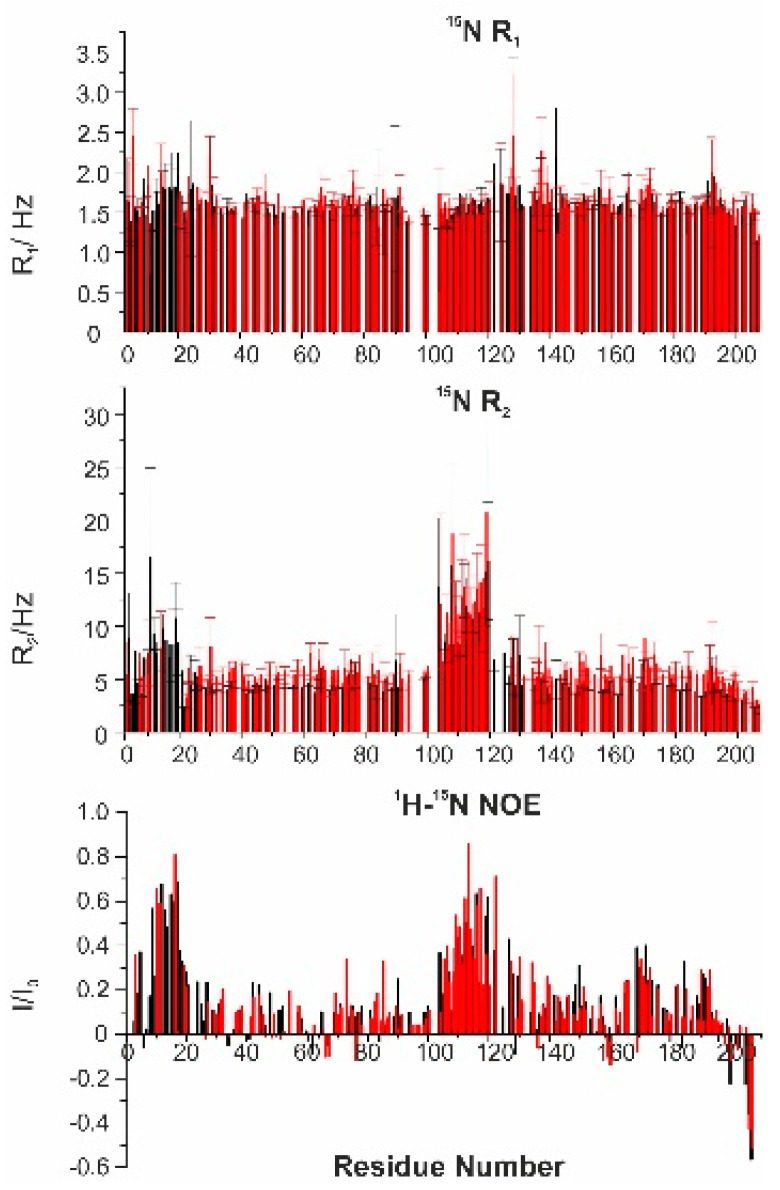
Nuclear relaxation data for backbone amide ^15^N obtained for CBP-ID4 (black) and the 1:1 complex CBP-ID4:E1A12S (red). From the top to the bottom, ^15^N longitudinal relaxation rates (R_1_, Hz), ^15^N transverse relaxation rates (R_2_, Hz) including proline residues, and ^1^H-^15^N NOE.

To investigate the dynamic properties of the 1:1 complex of CBP-ID4 and E1A12S, ^15^N R_1_, ^15^N R_2,_ and ^1^H-^15^N NOE were measured (Figure 8). The missing values in the ^15^N R_2_ plot are due to the broadening of the signals of the nuclei mostly affected by the interaction in the region of residues 2–28. The determined R_2_ values are slightly higher for the protein in the complex with respect to the isolated protein. The R_2_ values for the proline residues were measured using ^13^C-detected experiments [34] showing very little variation in their dynamic properties. Also, for CBP-ID4, the heteronuclear NOEs and the R_1_ values were almost unchanged.

In summary, the region of CBP-ID4 that is mainly perturbed upon the addition of E1A is the first partially populated helical fragment (2–25), the one with a net positive charge deriving from the presence of five arginine residues and no negatively charged ones, while the second helix which has no overall net charge (2 Arg and 2 Glu) is not significantly perturbed. The direct detection of the signals of proline residues also reveals that the long stretches of the polypeptide sequence, particularly rich also in serine and threonine residues, are not perturbed.

Overall, the effect of the interaction on the dynamics of the two proteins points to a slight increase in ^15^N R_2_ values spread all over the two polypeptides, with a higher increase in the regions showing α-helical propensity. This effect can be related to the decreased flexibility of the two proteins in the complex with respect to the two isolated forms as well as to contributions from exchange processes. Nevertheless, there are no indications of the occurrence of major changes in the conformation in any of the two proteins. This was confirmed by the circular dichroism analysis of the two separated proteins and of the complex (Figure S2) suggesting that the latter maintains an overall general disorder and flexibility typical of fuzzy complexes [49,50,51,52,53,54]. 

## 4. Discussion

The human AdV E1A12S protein is an intrinsically disordered protein of 243 amino acids. E1A is among the most extensively studied viral transcriptional regulators, however, despite a large number of studies about E1A interactions, the structural characterization at the atomic level of the entire protein was obtained only very recently exploiting NMR spectroscopy [2,5]. The NMR characterization unveils the high disorder of E1A12S, but with heterogeneous behavior in terms of structural and dynamic properties. This protein presents a significant α-helical secondary structure propensity in the region encompassing residues 12–22; the remaining part of the protein adopts flexible and extended conformations with many proline residues uniformly distributed along the polypeptide chain. More importantly, the NMR assignments available enable us to achieve high-resolution information on the mode through which E1A recognizes proteins of the host cell and, to this end, we decided to start by focusing on the interaction with CBP. While the entire E1A is amenable for NMR studies, the large size and complexity of CBP still prevent its investigation in the full-length form. However, two of its globular domains, the TAZ2 and the NCBD were found to interact with E1A, a quite surprising feature considering that they are separated by a long stretch of the polypeptide chain of more than 200 amino acids, the CBP-ID4. This fragment is thus expected to be involved in the interaction of E1A with CBP, even if its role remained elusive due to a lack of high-resolution NMR information in this region, which only recently became available [19]. Our data show that full-length E1A interacts with CBP-ID4 through an extended region of more than 80 amino acids in the N-terminal fragment. 

This region has a common characteristic: a high abundance of negatively charged residues as well as branched-chain hydrophobic ones. However, several fragments with peculiar features can be identified with an initial stretch (2–32) transiently adopting a helical conformation (12–22), followed by a more flexible one with a high abundance of proline residues (33–64), and a final part (65–88) that also features two aromatic-proline pairs (66–67 and 83–84). The NMR data obtained on E1A showed that this whole region was perturbed upon the addition of CBP-ID4, even if to different extents in the three parts mentioned above. It was previously reported that E1A uses multiple molecular recognition features (MoRFs) [55,56] that confer high specificity but low affinity to the interaction with target proteins, resulting in a stable high affinity interaction. The FXDXXXL motif (66–72) of E1A can interact with the CBP-TAZ2 and is also found in other viral and cellular proteins that bind CBP. This fragment is also involved in the interaction with CBP-ID4, and the latter might have an active role in promoting the interaction with CBP-TAZ2.

The picture is different when we look at the interaction from the point of view of CBP-ID4. The CBP-ID4 linker has proline residues uniformly distributed along the sequence, except for two regions that reveal two partially populated helical fragments. These two regions have different characteristics, the first one showing an overall positive electrostatic potential, with five arginine residues, while the second helix has no net charge and an overall hydrophobic surface. Thus, these two elements can provide specific binding modules for different interactions and the most positively charged one is an ideal target for E1A. Indeed, only residues in the first partially populated helix are perturbed upon the addition of E1A. Electrostatics must thus play a relevant role in the interaction. On the other hand, if electrostatics were the only driving force for interaction, the regions of E1A with the highest local negative charge, such as the poly-E/D region (^133^DDEDEEGEE^141^) or the (EP)_5_ region (^145^EPEPEPEPEP^154^) would be the most suitable ones to interact with a positively charged counterpart. Instead, the major perturbations are observed in the regions of E1A that share as a common feature the presence of negatively charged amino acids alternating with hydrophobic and bulky ones. The latter are thus expected to have a relevant role in stabilizing the interaction as well. The emerging picture is thus of a long stretch, essentially the first half of the primary sequence of E1A, that has a considerable affinity for the first partially populated helix of CBP-ID4. The latter, even if quite long (24 aa), cannot account for binding to the whole stretch of amino acids of the partner unless through fuzzy interactions of comparable affinity for the different patches of E1A. The presence of charged residues in IDPs has proven to be a prerequisite for the lack of a compact structure in proteins under physiological conditions [57]. In the present case, this is particularly striking: the high proportion of hydrophobic amino acids would tend to interact with each other unless a high abundance of residues with the same charge were present, causing local repulsion as well as contributing to solubility through solvent interactions. On the other hand, this amino acidic composition may be instrumental for recognition and binding to protein partners by allowing hydrophobic amino acids to engage in interactions with similar amino acids of the partner in synergy with complementary charges driving partner recognition. Electrostatic interactions can modulate the binding kinetics and the binding mechanism, helping the recognition of the target and promoting the interaction of hydrophobic structural elements. The NMR spectra of the E1A12S:CBP-ID4 complex at the different salt concentration indeed suggested that the first driving force of the interaction was electrostatic. The increase in the salt concentration weakened the binding (Figure S1), screening the favorable attraction between opposite charges, as in most electrostatic interactions [58]. 

The first helix of CBP-ID4 is preceded by CBP-TAZ2, a globular domain previously studied by NMR also in its interaction with an E1A short fragment [28]. CBP-TAZ2 is constituted by four α-helices separated by kinks harboring zinc(II) binding sites that stabilize this minimal fold. The first CBP-ID4 helix can thus also be considered an extension of the globular fold, as also identified in a construct characterized by X-ray (PDB ID 3IO2) [27]. The distinction between globular fold and flexible linker thus becomes quite labile as evident from this experimental investigation. 

**Figure 9:**
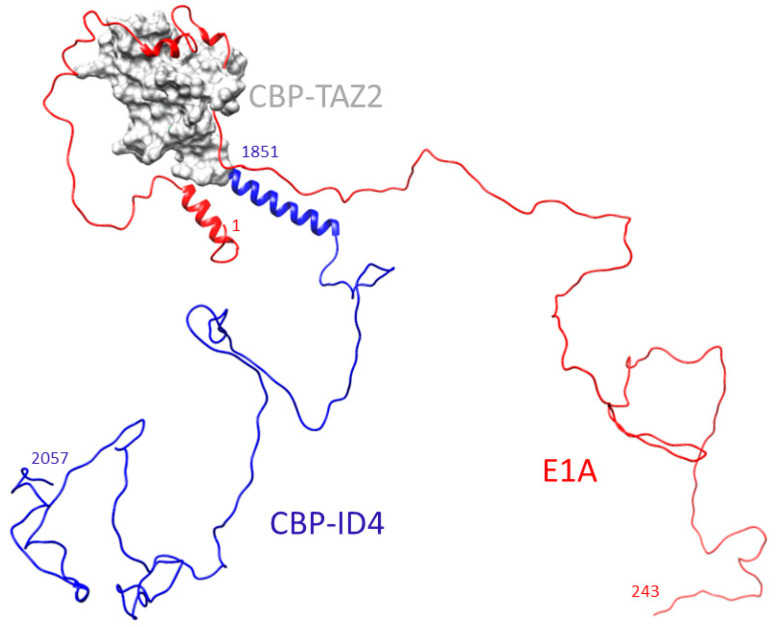
Cartoon describing one of the possible modes of interaction of E1A12S with the CBP-region comprising the TAZ2 domain and ID4. The structure of TAZ2 (grey surface) and of the fragment 53–91 of E1A is the first model of the PDB ID 2KJE entry (solution structure). The fragments 1–52 and 92–243 of E1A as well as the CBP-ID4 fragment were selected among the conformers of Flexible Meccano generated ensembles.

We can now frame our results in the context of the previous NMR investigations of the interaction between E1A and CBP-TAZ2: two short fragments of E1A (E1A_1–36_, part of the N-terminus and E1A_53–91_, containing part of CR1 and ten hydrophobic residues which are well conserved among E1A serotypes) were identified as interaction sites with CBP-TAZ2 [24]. In particular, the fragment E1A_53–91_ folds upon binding in a grove in the surface of CBP-TAZ2, with the hydrophobic amino acids as well as the two aromatic residues forming a tight interface between the two proteins (Figure 9). The fragment E1A_1–36_ instead interacts with a different region of CBP-TAZ2, towards the end of the C-terminal part of this domain. This is the region where the linker CBP-ID4 starts with the transiently populated helical conformation (Helix I) identified as involved in the interaction with E1A in the present study. Interestingly, the regions of E1A with high affinity for CBP-TAZ2 are also the ones that are mainly influenced by the interaction with CBP-ID4. The overall picture that emerges from these data is thus of a highly negatively charged module, the first part of E1A, that is able to bind not only to the first helix of ID4 but also to the whole, positively charged CBP-TAZ2, exploiting the many hydrophobic amino acids present to enhance the binding affinity once the electrostatic-driven recognition has been achieved. The highly flexible nature of E1A is thus instrumental to enable it to adapt to different forms of positively charged surfaces comprising the four helices of CBP-TAZ2 and the first helical region of CBP-ID4. This long negatively charged region of E1A has the potential to interact with several modules with complementary charge: it can also easily adapt to structurally heterogeneous and variable complementary surfaces. The hydrophobic amino acids present in this polyanion, combined with the plasticity and adaptability of this fragment enable sampling, in dynamic equilibrium and with comparable affinity, different conformations of the partner contributing to this fuzzy interaction. The affinities of the short fragments characterized by complementary charges, even if individually not so large, are likely to contribute to the affinity between E1A and this part of CBP in the full length protein, probably at the origin of how E1A competes with the multitude of functional interactions of CBP with other proteins of the host. Indeed, E1A has to target several modules of CBP which are functionally engaged with a variety of physiological partners. It is thus conceivable that E1A adopts all the possible strategies to interfere with CBP function, including binding to parts of its disordered linkers.

This study also shows how our idea of complex proteins as constituted by folded domains on one side and disordered segments on the other is just a simplification of a continuum between these two extremes. Indeed, CBP-ID4, always believed to be a “disordered linker”, harbors two partially populated α-helices that are quite extended, comprising about 25 amino acids each, separated by larger fragments of more disordered polypeptides. The globular domains flanking CBP-ID4 are actually not much longer: TAZ2 is about 92 AA [28] while NCBD is even smaller, comprising a total of 59 amino acids [59]. Moreover, the final part of the entire protein has always been considered as a flexible linker, namely CBP-ID5. Recently, it was characterized at atomic resolution and revealed a segment with helical propensity [21]. Thus, from TAZ2 to the end (1856–2442 AA), the two globular domains characterized up to now only comprise 20% of the CBP polypeptide chain. The remaining part hosts quite long regions with pronounced helical propensities and other segments with distinct amino acidic composition encoding functions that are only now beginning to be discovered. This shows the importance of such regions and the need to understand their role. The disordered regions of CBP-ID4 are rich in Pro and Ser/Thr residues, all amino acids promoting elongated conformations prone to post-translational modifications such as phosphorylation, which can change the overall charge of the molecule or the interaction propensity, adding a layer of complexity to the molecular mechanisms underlying the protein’s function. 

## 5. Conclusions

In this study, the interaction of the entire E1A12S protein with the ID4 fragment of CBP has been mapped at atomic resolution. A large, negatively charged patch of E1A was found to interact with the first, positively charged helical region of CBP-ID4. Several of the regions of E1A involved in this interaction were also found to interact with TAZ2, the globular domain preceding ID4. This highlights the versatility of E1A in recognizing different protein surfaces with a net positive charge also exploiting the many bulky hydrophobic amino acids that, thanks to the high flexibility of E1A, can adapt to different complementary surfaces and contribute to the binding affinity, potentially wrapping around this region of CBP. We can speculate that this could be a strategy used by the virus to compete with the many physiological interactions in which CBP is involved. Further studies are in progress to corroborate this hypothesis.

NMR spectroscopy has the potential of probing the more dynamic aspects of IDPs interactions, allowing the characterization at atomic resolution of highly dynamic complexes. This is particularly important to understand the specificity of IDPs towards their partner proteins, the potential interplay between different linear motifs, and in general, the regulatory mechanisms associated with multivalent interactions. It will be interesting to follow further progress in understanding these kinds of interactions as the NMR potential grows.

## Supplementary Materials

The following are available online at https://www.mdpi.com/2218-273X/10/11/1541/s1, Figure S1: Monitoring the interaction at increasing ionic strength, Figure S2: Circular dichroism analysis.
