# Supplementary material for: Adenoviral E1A Exploits Flexibility and Disorder to Target Cellular Proteins
Source: Biomolecules. 2020 Nov 11;10(11):1541. doi: 10.3390/biom10111541 (PMC7698142; doi:10.3390/biom10111541)
Supplement: Supplementary file 1 [file biomolecules-10-01541-s001.pdf]

Adenoviral E1A exploits flexibility and disorder to target cellular proteins

Maria Grazia Murrali, Isabella C. Felli and Roberta Pierattelli

Magnetic Resonance Center (CERM) and Department of Chemistry "Ugo Schiff", University of Florence, Via Luigi Sacconi 6, 50019 Sesto Fiorentino, Italy.

Supplementary information

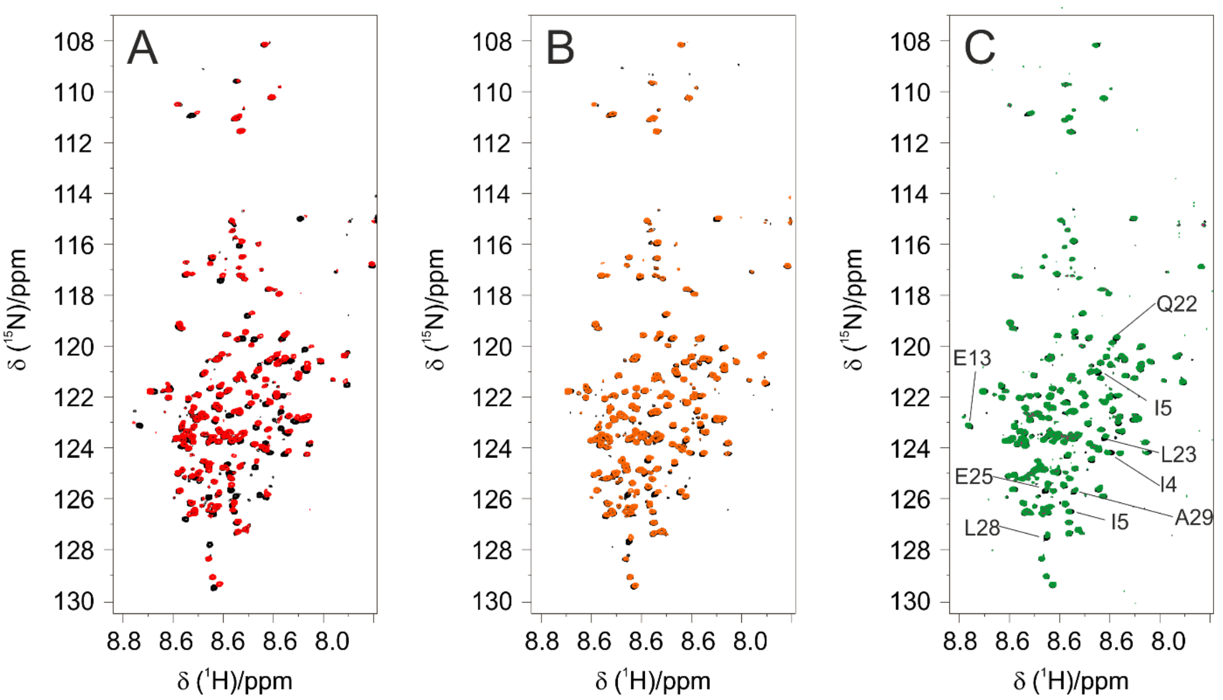

Figure S1. Monitoring the interaction at increasing ionic strength. (**A**) Comparison of ^{1}H-^{15}N BEST-TROSY experiments of isolated form of ^{15}N-E1A12S (black) and E1A12S:CBP-ID4 at 1:1 molar ratio (red) at 50 mM KCl; (**B**) comparison of ^{1}H-^{15}N BEST-TROSY experiments of isolated form of ^{15}N-E1A12S (black) and E1A12S:CBP-ID4 at 1:1 molar ratio (orange) at 150 mM KCl; (**C**) comparison of ^{1}H-^{15}N BEST-TROSY experiments of isolated form of ^{15}N-E1A12S (black) and E1A12S:CBP-ID4 at 1:1 molar ratio (green) 300 mM KCl. The assignment of selected peaks is also reported. All the experiments were acquired at 283 K, using a 22.3 T Bruker Avance III spectrometer equipped with a TCI CryoProbe™.

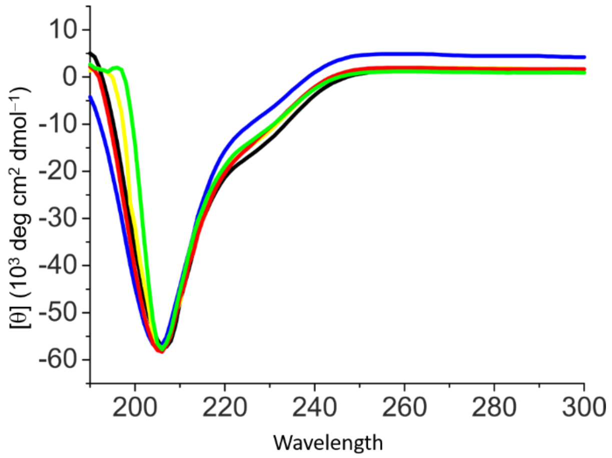

Figure S2. Circular dichroism analysis. Circular Dichroism spectra of E1A12S (black), ID4 (blue), E1A12S:CBP-ID4 1:0.5 complex (yellow), E1A12S:CBP-ID4 1:1 (red), E1A12S:CBP-ID4 1:2 (green).
